# Carbon dynamics in long-term starving poplar trees—the importance of older carbohydrates and a shift to lipids during survival

**DOI:** 10.1093/treephys/tpad135

**Published:** 2023-11-06

**Authors:** Juliane Helm, Jan Muhr, Boaz Hilman, Ansgar Kahmen, Ernst-Detlef Schulze, Susan Trumbore, David Herrera-Ramírez, Henrik Hartmann

**Affiliations:** Max Planck Institute for Biogeochemistry, Department of Biogeochemical Processes, Hans-Knöll-Str.10, Jena 07743, Germany; Department of Environmental Sciences–Botany, University of Basel, Schönbeinstr. 6, Basel CH-4056, Switzerland; Max Planck Institute for Biogeochemistry, Department of Biogeochemical Processes, Hans-Knöll-Str.10, Jena 07743, Germany; Department of Forest Botany and Tree Physiology, Laboratory for Radioisotopes, Georg-August University Göttingen, Büsgenweg 2, Göttingen 37077, Germany; Max Planck Institute for Biogeochemistry, Department of Biogeochemical Processes, Hans-Knöll-Str.10, Jena 07743, Germany; Department of Environmental Sciences–Botany, University of Basel, Schönbeinstr. 6, Basel CH-4056, Switzerland; Max Planck Institute for Biogeochemistry, Department of Biogeochemical Processes, Hans-Knöll-Str.10, Jena 07743, Germany; Max Planck Institute for Biogeochemistry, Department of Biogeochemical Processes, Hans-Knöll-Str.10, Jena 07743, Germany; Max Planck Institute for Biogeochemistry, Department of Biogeochemical Processes, Hans-Knöll-Str.10, Jena 07743, Germany; Max Planck Institute for Biogeochemistry, Department of Biogeochemical Processes, Hans-Knöll-Str.10, Jena 07743, Germany; Institute for Forest Protection, Julius Kühn-Institute, Federal Research Centre for Cultivated Plants, Erwin-Baur-Str. 27, Quedlinburg 06484, Germany

**Keywords:** bomb radiocarbon ^14^C, carbon allocation, CO_2_ efflux, non-structural carbon, O_2_ influx, stem respiration, tree girdling, ^13^C of respired CO_2_

## Abstract

Carbon (C) assimilation can be severely impaired during periods of environmental stress, like drought or defoliation, making trees heavily dependent on the use of C reserve pools for survival; yet, the dynamics of reserve use during periods of reduced C supply are still poorly understood. We used stem girdling in mature poplar trees (*Populus tremula* L. hybrids), a lipid-storing species, to permanently interrupt the phloem C transport and induced C shortage in the isolated stem section below the girdle and monitored metabolic activity during three campaigns in the growing seasons of 2018, 2019 and 2021. We measured respiratory fluxes (CO_2_ and O_2_), non-structural carbon concentration, the respiratory substrate (based on isotopic analysis and CO_2_/O_2_ ratio) and the age of the respiratory substrate (based on radiocarbon analysis). Our study shows that poplar trees can survive long periods of reduced C supply from the canopy by switching in metabolism from recent carbohydrates to older storage pools with a potential mixture of respiratory substrates, including lipids. This mechanism of stress resilience can explain why tree decline may take many years before death occurs.

## Introduction

Trees require sufficient carbon (C) to build up new biomass (including reproductive structures), fuel respiration, use C for defense and allocate C to storage pools (Chapin et al. 1990, Lambers and Poorter 1992, Sala et al. 2012). When the C supply from assimilation exceeds demand, trees can store substantial amounts of non-structural carbon (NSC). Those reserves may be used to maintain tree functions (e.g., respiration, osmoregulation, repair, biosynthesis of defense compounds) when the C supply is reduced below requirements, like during periods of harsh environmental conditions (e.g., Regier et al. 2009, Hartmann et al. 2013, Hartmann and Trumbore 2016, Zohner et al. 2019). Carbon storage compounds, including starch, sugars or lipids, provide an essential buffer against C shortage and play an essential role in the tree’s resilience capability (Hartmann and Trumbore 2016). Large NSC storage pools can be beneficial for the recovery of a tree after stress (e.g., insect herbivore defoliation, drought and fire) (Sala et al. 2010, Dietze et al. 2014, Piper and Paula 2020). The dynamics of reserve use and their availability during periods of reduced C supply in mature trees, over the short and long terms, are still poorly understood (Gessler and Treydte 2016, Hartmann and Trumbore 2016). For a more comprehensive understanding of C storage and remobilization dynamics in trees, studies over several years are needed to improve predictions of tree and forest resilience over time (McDowell 2011, Rosas et al. 2013, Gessler and Treydte 2016).

In order to gain insights into C reserve use under stressful conditions, one can artificially produce a lack of photo-assimilate supply via stem girdling. When removing a circumferential band of bark, phloem and cambium of a tree, the C supply from the canopy to the lower stem section is interrupted, and only upward water transport through the xylem is maintained. The stem section below the girdle is isolated from the rest of the tree above and is forced to use C reserves from within the stem or from the root system to maintain metabolic activity beneath the girdle. To date, empirical evidence supporting substrate shifts in trees is scarce, but see Fischer et al. (2015) and Wiley et al. (2019). It is still unclear whether and to what degree all types of reserve compounds, including sugars, starch and lipids, can be used as a respiratory substrate when C supply is limited. Plant lipid metabolism is far less studied due to the methodological challenges in quantifying neutral lipids (Fischer and Höll 1991, Hoch et al. 2003, Fischer et al. 2015), but progress has been made (see Grimberg et al. 2018, Herrera-Ramírez et al. 2021).

The simultaneous measurement of CO_2_ and O_2_ allows calculating the ratio of CO_2_/−O_2_, a useful indicator for the respiratory substrate identity (cellular level: respiratory quotient (RQ)). Respiratory substrates differ in their stoichiometric ratios of C:O:H and in their degree of oxidation. Thus, during respiration, quantities of O_2_ required as electron acceptor vary, depending on the respiratory substrate. During the breakdown of carbohydrates, one molecule of O_2_ is consumed for each molecule of CO_2_ released, resulting in RQ ~ 1, while for the breakdown of lipids, more oxygen is needed, resulting in RQ ~ 0.7. While RQ refers to the respiratory processes in the strict sense (i.e., measured at the mitochondrion), the apparent respiratory quotient (ARQ, Angert and Sherer 2011) may imply post-respiratory processes also (see Trumbore et al. 2013 for a summary), and this is the case when measured away from the mitochondrion, e.g., at the tree stem. More precisely, highly soluble CO_2_ can be transported away from the respiration site (e.g., Teskey and McGuire 2002, McGuire and Teskey 2004), or refixation mechanisms during the day (stem photosynthesis) (e.g., Pfanz et al. 2002, Wittmann et al. 2006) or during the night (phosphoenolpyruvatcarboxylase hereafter PEPC, hereafter) can fix CO_2_ locally and therefore reduce the CO_2_ efflux (*E*_CO2_) to the atmosphere, leading to ARQ values <1 (Angert et al. 2012, Hilman and Angert 2016). However, the potential role of fixation via PEPC has been investigated mainly in the leaves and young green twigs of C3 plants (Berveiller and Damesin 2008), but this might be relevant as a mechanism of local CO_2_ removal, as high-potential PEPC activity has been measured in the stem wood of mature beech trees (Helm et al. 2023).

Sugars, starch and lipids can also be distinguished by their C isotope signals of respired CO_2_ (δ^13^C) (Gleixner et al. 1993, Cernusak et al. 2003, Bowling et al. 2008, Brüggemann et al. 2011). Former studies showed that 2-year old oak saplings shifted the substrate for respiration from recently fixed carbohydrates to starch reserves (below the girdle) after girdling (Maunoury-Danger et al. 2010), which was deduced from a δ^13^C enrichment of CO_2_ respired by stems (Brugnoli et al. 1988, Tcherkez et al. 2004). In young *Pinus sylvestris* trees, reducing C assimilation by experimental shading triggered a shift from carbohydrate-dominated respiration to almost pure lipid-based respiration, indicated by lower δ^13^CO_2_ as well as lower RQ (Fischer et al. 2015). The δ^13^C signal can also reflect environmental conditions (stomatal closure to avoid water loss), as e.g., a change toward a more enriched δ^13^C signal could be explained by expected changes in photosynthetic discrimination (Farquhar et al. 1989, Högberg et al. 1995).

To enhance our understanding of NSC dynamics in trees, it is important to know how long these C reserves can be stored and how fast they can be used. The bomb radiocarbon (^14^C) approach allows determination of the mean age of C assimilated by a plant, and thus can be used to estimate the age of substrates used for respiration by calculating the amount of time elapsed between fixation and use and the time trees take to tap into their long-term reserves (Levin et al. 2010, Trumbore et al. 2016). Amazonian tree stems below the girdle mobilized ~ 5-year-old C for respiration within 1 month of girdling and decade-old C ~1-year post-girdling (Muhr et al. 2018). NSC age of stump sprouts (*Acer rubrum*) regenerated following harvesting was maximum 17 years (Carbone et al. 2013) and maximum 16-year-old C was used for new fine root growth after hurricane damage in a seasonally dry tropical forest (Vargas et al. 2009).

**Figure 1:**
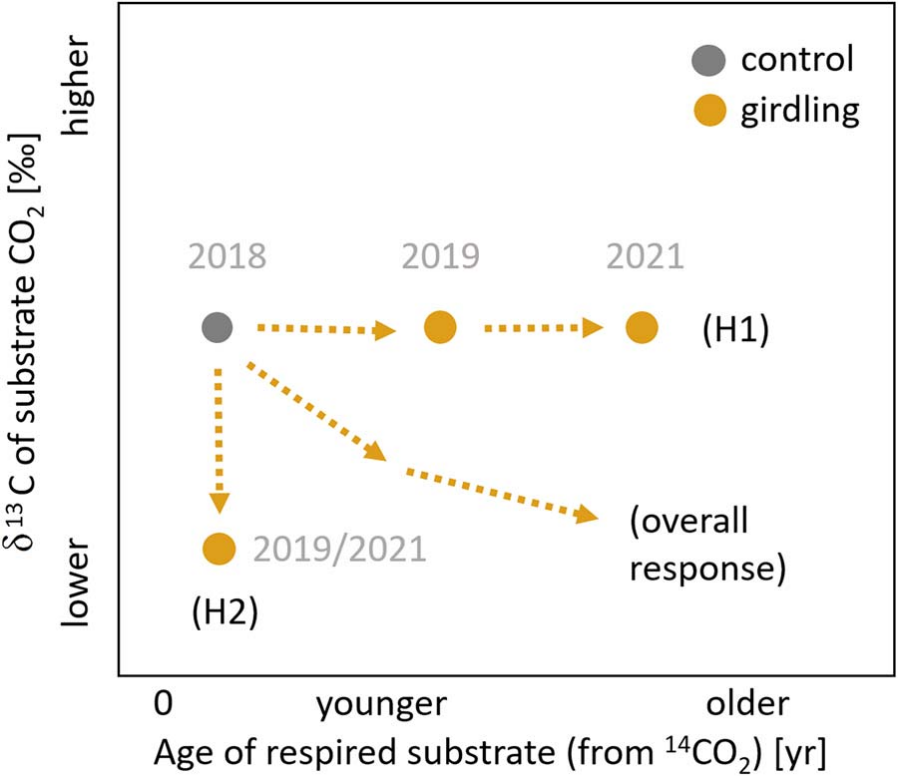
Graphical representation of the expected 3-year pattern of C reserve mobilization in mature poplar trees after girdling. Two hypotheses related to C reserve use (H1) and substrate identity (H2) are presented together with the expected overall response.

In our study, we investigated the responses of mature poplar trees (*Populus tremula* L. hybrids) to reduced C supply to stem sections. This species is a very common and fast-growing tree species that is known to store, besides sugars and starch, substantial amounts of lipids (Hoch et al. 2003). We acknowledge here the potential effect of root grafting during starvation, as C transfer between trees has been reported in mature poplar trees (DesRochers and Lieffers 2001, Fraser et al. 2006, Jelínková et al. 2009) and could compensate for the lack of photo-assimilates. We investigated how the reduced C supply of recent photo-assimilates via girdling affects the respiratory substrate use and mobilization of storage pools in the isolated stem section. In particular, we tested the following hypotheses (Figure 1):

(H1) After the disruption of the supply of photo-assimilates, poplar trees initially mobilize NSCs (decrease in NSC concentration), increasingly digging into older C reserves (increase in Δ^14^C).

(H2) Lipids contribute to metabolism maintenance during starvation, indicated by the progressive mobilization and metabolization of lipids as starvation proceeds (decline in ARQ ratios and lower δ^13^CO_2_ signal).

## Materials and methods

### Study site and girdling treatment

**Figure 2:**
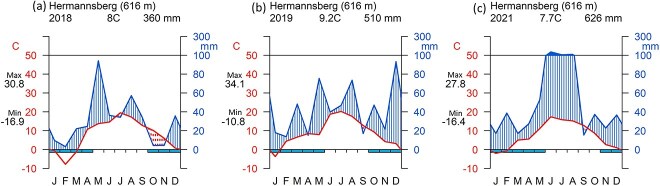
Walter Lieth climate diagram of the study site Hermannsberg (Germany) in (a) 2018, (b) 2019 and (c) 2021. Average annual temperature (°C) and annual precipitation (mm) are shown. Max: mean of the maximum temperatures of the warmest month. Min: mean of the minimum temperatures of the coldest month.

The study site is located in the Thuringian Forest, Germany (50°42′50″N, 10°36′13″E, site elevation 616 m a.s.l., north slope). Mean annual temperature is ~7 °C and the mean annual precipitation is 800–1200 mm (Bouriaud et al. 2016). Soil was formed on a volcanic bedrock. Our measurements were carried out in the growing seasons (May–September) of 2018 and 2019. We included a short measurement campaign in 2021, as most of the girdled trees were still alive (with a reduced canopy leaf area; visual inspection) after 3 years. Meteorological information was available from a weather station nearby, however, not directly at the north slope. Annual precipitation was 360 mm (2018), 510 mm (2019) and 626 mm (2021), respectively (Figure 2). The average annual temperature at our site was 8 (2018), 9.2 (2019) and 7.7 °C (2021), respectively (Figure 2)*.* In 2018 and 2019, extreme summer drought affected central Europe (Bastos et al. 2021). In 2018, we selected 12 mature poplar trees (*Populus tremula* L. hybrids, ~60 years-old) at minimum 3-m to maximum 18-m distance to the neighboring tree, which were free of obvious signs of injury or disease, with easily accessible stems and stem diameter at breast height between 29 and 42 cm (Table S1 available as Supplementary data at *Tree Physiology* Online). Those trees were growing on terraces that were formerly used for agriculture, which afterward became grassland and then forest. On 4 July 2018 (DOY 185), 6 of the 12 trees were randomly chosen and girdled by carefully removing a ~4-cm-wide circumferential band of bark, cambium and phloem at ~1.5 m height above ground (Picture S1 available as Supplementary data at *Tree Physiology* Online). All stem measurements were made below the girdle (for girdled trees). For an overview of the different measurements and the timing of sampling, see Table S2 available as Supplementary data at *Tree Physiology* Online. As we could not see any signs of wound repair/cambium regrowth at the girdling band, nor any sprouting over the time of measurements, we assume a continuous interruption of the phloem transport pathway over 3 years.

### Stem CO_2_ efflux and O_2_ influx measurements

We installed automated measurement chambers for quantifying stem CO_2_ efflux (*E*_CO2_, hereafter) and O_2_ influx (*I*_O2_, hereafter) (Helm et al. 2021). The highly autonomous low-cost chamber-based measurement device was installed at a height of ~1.3 m, i.e., below the girdling on the girdled trees (Picture S2 available as Supplementary data at *Tree Physiology* Online). In 2018, chambers were installed on all trees and measurements were conducted from 5 May to 20 September. Due to limited capacity, in 2019, chambers were installed on three control and three girdled trees from 2 June to 2 September. From 2 July to 3 August 2021, chambers were installed on four control and four girdled trees. Chambers were installed on the north side of the trees and were covered with reflective foil to prevent heating from direct solar radiation. For details about the chamber set-up and sensor specifications, see Helm et al. (2021). Configuration settings were based on a repeated closed chamber mode with 45-min measurement cycles (CO_2_ and O_2_ raw data were recorded every 10 s). Each cycle was followed by a 15-min flushing period of the chamber with ambient air before a new measurement cycle started.

As a general requirement, O_2_ as a non-trace gas needs to be corrected for the dilution effect of changing H_2_O and CO_2_ concentrations (Helm et al. 2021); therefore, we used the relative humidity sensor integrated in the COZIR non-dispersive infrared absorption sensor (Gas Sensing Solution GSS, Cumbernauld, UK) for the correction. Relative humidity was converted to [H_2_O] using the Magnus formula (see Helm et al. 2021).

Measurements of CO_2_/−O_2_ headspace concentrations over time are subsequently used to calculate the CO_2_ and O_2_ fluxes. To this end, the linear increase of CO_2_ and decrease of dilution-corrected O_2_ concentrations of the first 20 min were used after removing the first 5-min period following flushing to avoid the influence of pressure fluctuations:


(1) \begin{equation*} \mathrm{Flux}=\frac{\Delta C}{\Delta t}\times \frac{\ V}{A}\times \frac{P}{R\times T}, \end{equation*}

where Δ*C*/Δ*t* is the change in gas concentration over time (p.p.m. s^−1^) for CO_2_ and O_2_ (absolute value), respectively, *V* is the volume of the chamber (m^3^), *A* is the stem surface area (0.0028 m^2^), *P* is the barometric pressure (kPa; from LuminOx sensor), *R* is the molar gas constant (0.008314 m^3^ kPa K^−1^ mol^−1^) and *T* is the temperature (Kelvin). Volumes of the stem chambers ranged from 70 to 105 cm^3^ and were determined after installation by injecting water with a calibrated syringe into the chamber headspace. To allow air-bleeding from headspace, we inserted two syringe needles into the chamber headspace; one to inject water, and the other to vent air from the chamber (Table S1 available as Supplementary data at *Tree Physiology* Online).

### Sensor calibration

In 2018, CO_2_ sensors initially were calibrated every 6 weeks. Upon noticing substantial sensor drift beyond 3 weeks since calibration (Helm et al. 2021), we excluded all data recorded >3 weeks since last calibration, and from 2019 onward, sensors were calibrated every ~3 weeks. For O_2_ sensors, electronic storing of calibrated parameters was not possible; therefore, the stability/validity has been checked regularly by evaluating possible drift (predefined limit of the slope: 1 ± 0.03) using different reference gas concentrations (Westfalen AG, Münster, Germany). For more in-depth information about the calibration procedure and calibration unit, see Helm et al. (2021).

### Sap flow rate

Sap flux density (l cm^−2^ h^−1^) was monitored during the growing season 2018 (May–September), only. We measured the sap flow with Sap Flow Meter SFM1 sensors from ICT International installed below the glass flask chamber (see ^14^C and ^13^C signatures of respired CO_2_) at ~0.5 m stem height. Sap flux density was recorded every 20 min and was converted to sap flow rate (l h^−1^) by using the software Sap Flow Tool (ICT International, University Ghent). Sapwood depth was assumed to be 50% of the xylem radius.

### Non-structural carbohydrate analysis

The effect of girdling on the storage reserves in the sapwood was evaluated by seasonal NSC measurements of stem cores. In 2018, we sampled the stem cores twice a year, before the girdling (DOY 172) and 90 days after the girdling (DOY 262). In 2019 and 2021, we sampled once a year (DOY 147 and DOY 195). Stem cores from all 12 trees were immediately placed in a cooler (0–5 °C) for transport to the laboratory, where they were dried for 72 h at 60 °C within 4 h after the core collection in the field. Cores were sanded with sandpaper to facilitate the identification of annual rings under a light microscope (Stemi 2000-C, Carl Zeiss Microscopy GmbH, Göttingen, Germany). Defining the outermost (most recent) annual ring as ring 1, we then cut the cores into pieces consisting of rings 1–6 (without bark) and 7–14. Wood material was ground to a fine powder in a ball mill. Aliquots of 30 mg of homogenized wood material were analyzed for concentrations of sugars (glucose, fructose and sucrose) and starch according to protocols S1 and S2 from Landhäusser et al. (2018). In short, ethanol (80% v/v) was used as the solvent for sugar extraction. After vortexing for 1 min, incubating at 90 °C for 10 min and centrifuging at 13,000*g* for 1 min, supernatants were analyzed by a high-performance liquid chromatography coupled to a pulsed amperometric detection (HPLC-PAD). Concentrations are expressed in glucose equivalents per dry wood mass. Starch was extracted from the remaining pellet from the soluble sugar extraction using two digestive enzymes: alpha-amylase and amyloglucosidase (Sigma-Aldrich). The glucose hydrolysate was measured by HPLC-PAD.

### ^14^C and ^13^C signatures of respired CO_2_

We repeatedly collected gas flask samples for δ^13^CO_2_ and Δ^14^CO_2_ measurements by means of additional stem chambers that were installed in close proximity below the respiration chamber (Picture S2 available as Supplementary data at *Tree Physiology* Online). A glass flask chamber consisted of polypropylene plate equipped with three connectors for sampling flasks and a foam frame (2.4 cm thick; ^14^C neutral material) placed between the stem and the plate to ensure airtight sealing. Chambers for sampling isotopes were installed temporarily for sampling campaigns using four rachet straps for fastening the chamber on the stem. Three flasks were connected to the chamber and opened. Each of these incubation periods lasted ~1 week to ensure sufficient amounts of CO_2_ for ^13^C and ^14^C analyses and for the establishment of steady-state conditions. Then, the flask inlets were closed and the glass flasks removed from the stem. The sampling flasks were custom-built, made of glass and had a volume of 115 mL. Glass flasks were evacuated prior to sampling and the inlets were equipped with a Louwers O-ring high-vacuum valve (Louwers H.V. glass valves, Louwers Glass and Ceramic Technologies, Hapert, The Netherlands) (Muhr et al. 2018). We conducted three pre-girdling samplings. Following girdling, sampling took place at approximately monthly intervals from July to October in the same year, from June to September in 2019 and from July to September in 2021. Leaks in the field or problems during extractions repeatedly resulted in smaller number of replicates than intended (*n* = 6 for control and girdling each) (Tables S3–S5 available as Supplementary data at *Tree Physiology* Online for ^13^C and ^14^C samplings). Flask samples were brought to the laboratory at the MPI-BGC in Jena for analysis.

For Δ^14^C of CO_2_, gas samples (~0.5 mg of C) were cryogenically purified, graphitized and analyzed with an accelerator mass spectrometer (Lowe 1984, Vogel et al. 1984, Steinhof et al. 2017, Muhr et al. 2018). Radiocarbon data are reported as Δ^14^C (‰), i.e., the per mil deviation from the ^14^C:^12^C ratio of oxalic acid standard in 1950. Accounting for any mass-dependent fractionation effects, Δ^14^C is corrected to a δ^13^C value of −25‰ (Stuiver and Polach 1977). Detailed calculation can be found in Trumbore et al. (2016). The ∆^14^C of any given sample can be used for estimating the ‘age’ of respired CO_2_ by calculating the difference to the atmospheric Δ^14^C of the study site at the time of sampling. The local atmospheric Δ^14^C record between 1950 and 2019 was estimated previously by Hua et al. (2022) (northern hemisphere zone 1) and Hilman et al. (2021). We added to this record an estimation for 2021 atmospheric Δ^14^C by analyzing local annual plants (*Rumex* spp., *Stachys* spp.), which is assumed to fix the majority of its C from the 2021 growing season atmospheric CO_2_. Samples with Δ^14^C values clearly below atmospheric Δ^14^C (<5‰) were discarded, as those samples might reflect the influence of CO_2_ from the local fossil sources.

We used the following formula to estimate the mean age of respired CO_2_ (year) according to Hilman et al. (2021):


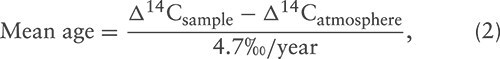


where Δ^14^C_sample_ is the measured value from the gas sample, Δ^14^C_atmosphere_ is the signature of the current atmospheric CO_2_ and 4.7‰ is the mean annual decline in atmospheric Δ^14^C. The estimate for the atmospheric Δ^14^C during the growing seasons was +2.3‰ (2018), −2.4‰ (2019) and −5.4‰ (2021), respectively.

For δ^13^CO_2_ measurement, two aliquots (50 μL) from each gas sample were analyzed with an isotope ratio mass spectrometer (Delta+ XL; Thermo Fisher Scientific, Bremen, Germany) coupled to a modified gas bench with a Conflow III and GC (Thermo Fisher Scientific). The δ^13^CO_2_ samples were analyzed against a laboratory air standard on the Vienna Pee Dee Belemnite scale realized by the Jena Reference Air Set-06 (Wendeberg et al. 2013). The values obtained were corrected using the Davidson equation (Davidson 1995) to account for fractionation effects:


(3) \begin{equation*} \frac{C_{\mathrm{s}}\left({\delta}_{\mathrm{s}}-4.4\right)-{C}_{\mathrm{a}}\ \left({\delta}_{\mathrm{a}}-4.4\right)}{1.0044\ \left({C}_{\mathrm{s}}-{C}_{\mathrm{a}}\right)} ,\end{equation*}

where *C*_s_ is the CO_2_ concentration of respired CO_2_ in the flask (p.p.m.), *δ*_s_ is the isotopic composition of respired CO_2_ (‰), *C*_a_ is the ambient air concentration of CO_2_ (assumed 400 p.p.m.) and *δ*_a_ is the isotopic composition of ambient air (assumed to be −9‰).

Besides δ^13^C of respired CO_2_, we also measured δ^13^C of soluble sugars and neutral lipids following a modified protocol (Bligh and Dyer 1959, White et al. 1979) and liquid chromatography (Schwab et al. 2019). Increment cores from all 12 trees were extracted at breast height using a standard 5.15 mm diameter increment borer (Haglöf Company Group, Sweden) in 2021 (DOY 236, rings 1–14). Wood material was ground to a fine powder in a ball mill (MM 400, Retsch, Haan, Germany), and in a next step, was phase-separated: water-soluble C was analyzed as a proxy for soluble sugars, and C extractable in the methanol:chloroform solution (total lipids) was transferred to silica gel column. The lipids that eluted by chloroform were regarded as ‘neutral’ and were analyzed (for further details, see Method S1 available as Supplementary data at *Tree Physiology* Online). Aliquots from the extractions were put into tin cups, dried and afterward the measurement was performed with a Finnigan MAT DeltaPlus XL EA-IRMS (ThermoFinnigan GmbH, Bremen, Germany), which was coupled to an autosampler (Koppenaal et al. 1991).

### Quantification of neutral lipids in stem woods

For the visualization and quantification of lipids, we took stem cores in 2021 (DOY 147) from three randomly selected trees from each treatment. To quantify neutral lipids in the stem wood, we used a histological method based on the protocols proposed by Mehlem et al. (2013) and Herrera-Ramírez et al. (2021). We took histological slides (30 μm thick) from the first 3 cm, from bark to pith. The slices were washed with distilled water and were then placed in a Petri box. Wood histological slices were stained with Oil Red O (ORO) to visualize the neutral lipids. The ORO stock solution was prepared by adding 2.5 g of ORO to 400 mL of 99% (vol/vol) isopropyl alcohol and by mixing the solution for 2 h at room temperature. The ORO working solution was prepared by adding 1.5 parts of ORO stock solution to one part of distilled water, shaking it for 5 min, letting it stand for 10 min at room temperature and filtering it through a 45-μm filter to remove the precipitates. The ORO working solution was added into the Petri box until completely covering the wood slices. We closed the Petri box to avoid drying and precipitating of the ORO solution and let the sample incubate for 20 min at room temperature. Then, we rinsed the samples with running distilled water for ca 15 min, changing water every 5 min. The histological slices were mounted on glass slides using water as a mounting medium and were placed under a coverslip. We took pictures of each histological slice within 1 h after mounting them on the glass slide. After that time, water started to dry out and the ORO solution started to precipitate. Panoramic photos of the wood slides were taken using an optical digital microscope with large depth of field (Keyence, VHX-6000, USA) at ×500 magnification.

We used the pictures to quantify the percentage of the aerial surface covered by neutral lipid droplets using ImageJ (Schneider et al. 2012). We quantified the percentage of lipid coverages in small regions of interest (ROIs) of 0.25 mm^2^ randomly generated by the automatic script used for Image J (Anexx 1). We divided the images in sections corresponding to 3 mm of wood counted from bark to pith, and in each 3 mm wood section, we measured 50 ROIs, leading to a total of 500 ROIs along the 3 cm of wood. We estimated the percentage of the aerial surfaced covered by neutral lipids in the wood as the average between all the measured ROIs along the wood sample.

### Potential PEPC activity in woody tissue

For potential PEPC activity measurements, we collected stem cores from all 12 trees in August 2019 (DOY 236). Cores were immediately frozen in liquid nitrogen in order to avoid any further metabolic activity, transported to the laboratory and stored at −80 °C freezer. We cut the first 2 cm of stem material (bark to xylem) and ground the wood to a fine powder with a mortar and pestle in liquid nitrogen. A discontinuous assay was performed following the steps of Bénard and Gibon (2016) in order to quantify potential PEPC activity. We used 20 mg of woody tissue material. All pipetting steps were performed using a 96-head robot (Hamilton Star). Aliquots, together with 500 μL of extraction buffer, were shaken for extraction. Extracts were centrifuged for 7 min (3000*g*, 4 °C) before the extracts were diluted by a factor of 2000 (w/v). The NAD^+^ standards were prepared in the before-mentioned extraction buffer (ranging from 0 to 1 nmol per well). Afterward, those standards and the diluted extracts were incubated for 20 min in a 20 μL medium (100 mM Tricine-KOH pH 8.0, 20 mM MgCl_2_, 1 unit mL^−1^ malate dehydrogenase, 10 mM NaHCO_3_, 0.1 mM NADH, 1% w/v polyvinylpyrrolidone, phosphoenolpyruvate 0 (blanks) or 2 mM (maximal activity)). In order to stop the reaction, 0.5 M HCl (20 μL) was used. In order to destroy NADH, the 96-well microplate was sealed and incubated for 10 min at 95 °C. In a next step, the microplate had to acclimate to room temperature, and a neutralization step with NaOH 0.5 M (20 μL) and 0.2 M Tricine-KOH followed to adjust the pH to 9.0. Together with 6 units mL^−1^ alcohol dehydrogenase, 100 mM Tricine-KOH pH 9.0, 4 mM EDTA, 0.1 mM PES, 0.6 mM MTT and 500 mM ethanol, NAD+ was quantified. The absorbance at 570 nm was measured at 30 °C in a filter-based microplate reader (SAFAS MP96). To calculate the amount of NAD^+^ formed during the first step of the assay, the reaction rates (mOD min^−1^) were used. For further details, see Bénard and Gibon (2016).

### Statistics

All analyses were performed using R software (R Development Core Team 2019). We used R package climatol for Walter—Lieth climate graph. We used pad function from the padr package for linear interpolation of the flux data to fill flux data gaps shorter than 2 h. Flux data were discarded if *R*^2^ of the slope of the linear regression was <0.96 and relative humidity >99% (after filtering, 2018: 89%; 2019: 81%; 2021: 65% used). The ARQ ratio was calculated as the slope of changing CO_2_ concentration over time divided by the negative slope of changing O_2_ concentration over time (slope CO_2_/−slope O_2_). For ARQ values, we applied an outlier removal function, accepting only ARQs between 25% quantile − 1.5 * interquartile range (IQR) and 75% quantile + 1.5 * IQR. Data were averaged over 6-h time intervals (net efflux of CO_2_ (*E*_CO2_), net influx of O_2_ (*I*_O2_) and ARQ) for raw data plotting. We computed daily mean values only when the data for the whole 24-h period exist.

We used the lme function (nlme package; Pinheiro et al. 2017) to perform linear mixed-effect models. We analyzed if the treatment influenced *E*_CO2_ and *I*_O2_, ARQ, NSC, δ^13^CO_2_ and Δ^14^CO_2_ month-wise (in 2018, 3-week-average). Treatment was considered as a fixed factor, while tree, and if applicable, sensor ID, to account for the effect of different sensors being installed in chambers across years, were considered as random factors. An autocorrelation structure was included into the models to account for temporal correlation. The model’s normality of residuals was checked visually (quantile–quantile plots). All results were expressed as mean ± standard deviation (SD).

## Results

### CO_2_ efflux, O_2_ influx and ARQ

**Figure 3:**
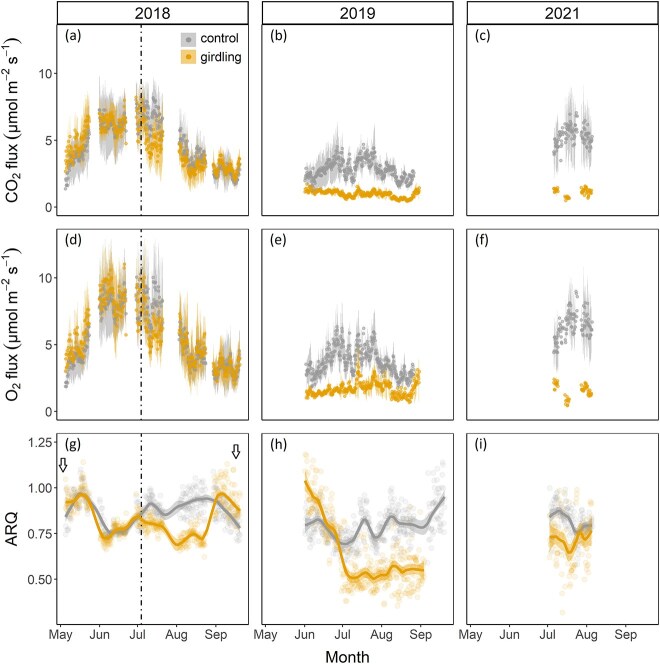
Upper panels: (a–c) CO_2_ efflux (*E*_CO2_) measurements (6 h mean ± SD) in 2018, 2019 and 2021. Middle panels: (d–f) O_2_ influx (*I*_O2_) measurements (6 h mean ± SD, absolute values) in 2018, 2019 and 2021. Lower panels: (g–i) ratio of CO_2_ efflux to O_2_ influx (ARQ) in 2018, 2019 and 2021 with a LOESS smooth (span = 0.4). Arrows indicate budburst before May and leaf-fall in September. The time of girdling is indicated by the vertical dashed line. All values are 6 h mean (*n* = 12 (2018), *n* = 6 (2019) and *n* = 8 (2021)).

The *E*_CO2_ and *I*_O2_ during the pre-girdling period did not differ between treatments (*P* = 0.48 and *P* = 0.53, for *E*_CO2_ and *I*_O2_, respectively, Figure 3). After the girdling event in 2018, a significant difference was observed in *E*_CO2_ between treatments for the measurement period in August (*P* < 0.01). One year after girdling, control and girdled trees differed significantly (*P* = 0.03 and *P* = 0.02, for *E*_CO2_ and *I*_O2_, respectively) with a marked decline of *E*_CO2_ and *I*_O2_ in girdled trees. However, in 2019, fluxes in control trees were also ca 40% lower than in 2018. Daily maximum values of 6.7 (control) and 2.4 (girdling) μmol m^−2^ s^−1^ were recorded for *E*_CO2_, while for *I*_O2_, daily maximum values reached 9.7 (control) and 2.9 (girdling) μmol m^−2^ s^−1^ in 2019. In 2021, differences between treatments increased (*P* < 0.001, *P* < 0.0001 for *E*_CO2_ and *I*_O2_). Control fluxes were twice as high as in 2019, roughly the same as in 2018. Differences between *E*_CO2_ and *I*_O2_ were significantly different in all 3 years (*P* < 0.001).

Before the girdling, the ratio of *E*_CO2_ to *I*_O2_ did not differ between treatments (*P* = 0.4) with daily mean ARQ values (±SD) of 0.85 ± 0.1 (control) versus 0.84 ± 0.1 (intended girdling; Figure 3). After the girdling event in 2018, daily ARQ values of the two treatments in August differed significantly (*P* = 0.02) with daily mean ARQ values of 0.93 ± 0.03 (control) versus 0.72 ± 0.02 (girdling). In 2019, a treatment effect was visible in the mean ARQ values (*P* < 0.01), with 0.77 ± 0.07 (control) versus 0.63 ± 0.2 (girdling) from July until the end of August. In June 2019, ARQ was higher in girdled than in control trees. In summer 2021, mean ARQ values did not differ significantly (*P* = 0.4) with 0.84 ± 0.1 (control) versus 0.78 ± 0.2 (girdling).

### Sap flow rate and ARQ in 2018

**Figure 4:**
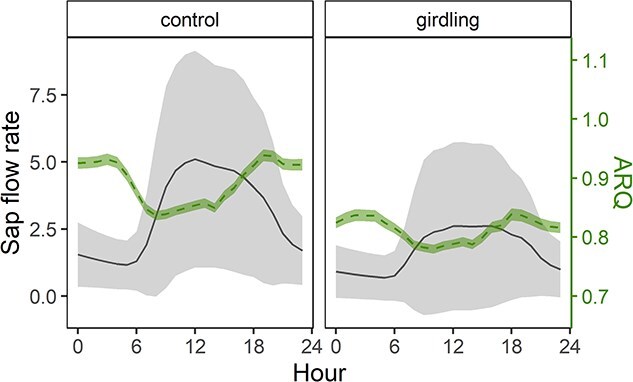
Subdaily values of the ARQ and sap flow rate (±SD) for control and girdled trees, respectively (data pooled from 5 July to 30 September 2018).

Sap flow rate (l h^−1^) clearly decreased after the girdling event (Figure S1 available as Supplementary data at *Tree Physiology* Online). When looking at the daily patterns of ARQ in 2018, the ratio was significantly higher during the night (~8 p.m.—~4 a.m.; 0.93 for control, 0.83 for girdling) compared with daytime (~8 a.m. to ~ 4 p.m.; 0.85 for control and 0.79 for girdling) when the sap flow rate is maximal (Figure 4). Negative correlation was found between the ARQ and sap flow rates (Pearson’s correlation, *r*^2^ = −0.57, *P* < 0.01 for both control and girdling).

### Non-structural carbohydrates and neutral lipids

**Figure 5:**
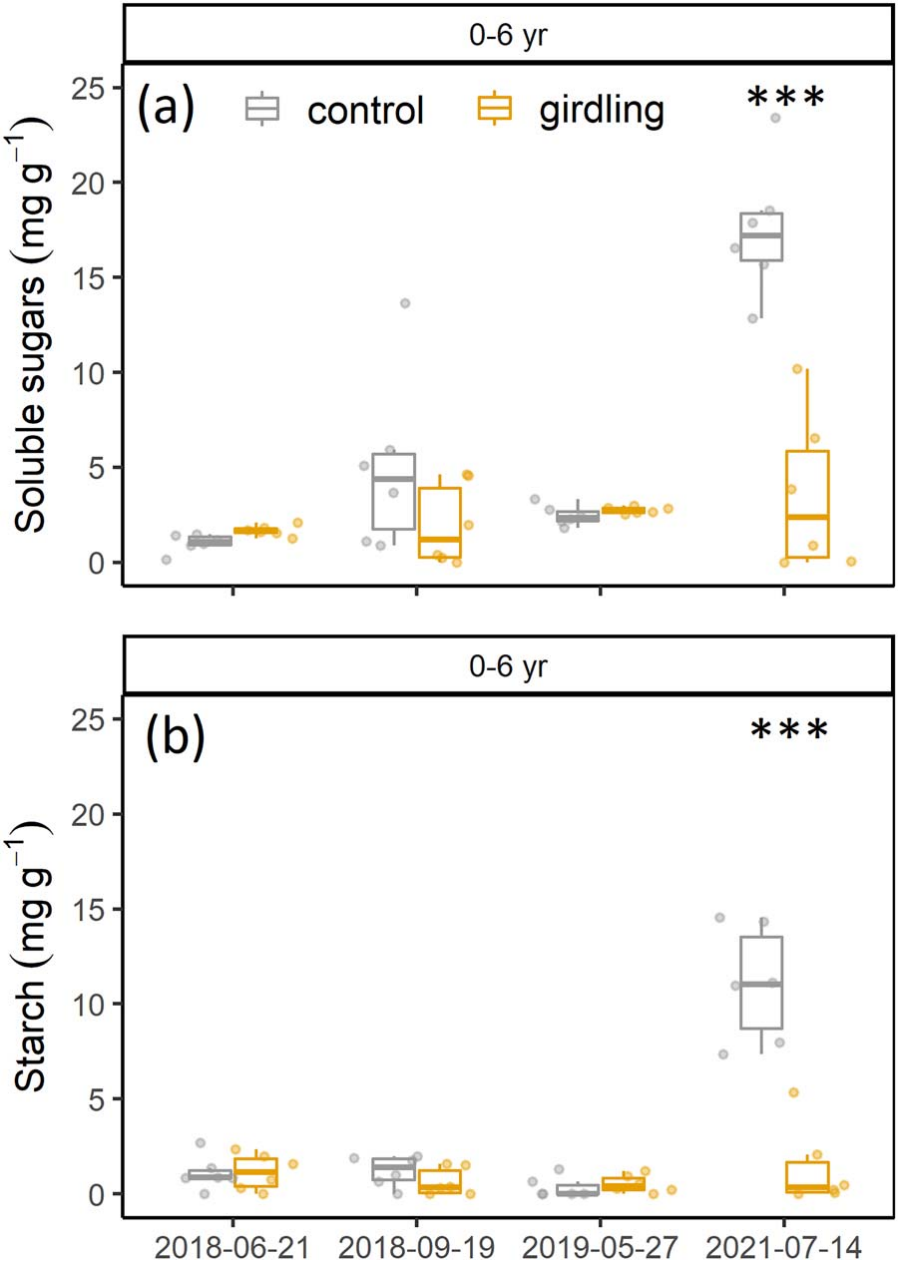
(a) Soluble sugar (glucose, fructose and sucrose) concentration (mg g^−1^) and (b) starch concentration (mg g^−1^) before girdling (21 June 2018) and three time points after girdling, extracted from stem cores to a depth of ring 6. Box whisker plots present the median, lower (25th) and upper (75th) percentiles, minimum and maximum values.

Pre-girdling sampling of the outer stem segment (0–6 years) showed no differences in the soluble sugar concentration of the xylem (glucose, fructose and sucrose; mg g^−1^) (*P* = 0.3) and starch concentration (mg g^−1^) between treatments (*P* = 1.0; Figure 5). In 2018 and 2019, the starch concentration was <2 mg g^−1^, independent of treatment. Soluble sugar concentration increased from 1.0 to 5.1 mg g^−1^ after the growing season (September 2018) in control trees. Finally, in 2021, the soluble sugar and starch concentrations varied significantly between treatments (*P* < 0.001 and *P* < 0.001, respectively), with a mean soluble sugar concentration of 17.5 ± 3.5 mg g^−1^ and a mean starch concentration of 11.0 ± 3.0 mg g^−1^ for control trees, while for girdled trees, the soluble sugar and starch concentrations remained low (3.6 ± 4.2 and 1.4 ± 2.2 mg g^−1^, respectively). The concentrations of soluble sugars and starch in the second stem segment to a maximum depth of ring 14 did not show significant differences in the concentrations in all 3 years (Figure S2 available as Supplementary data at *Tree Physiology* Online).

Neutral lipids, analyzed in 2021, were 0.76 ± 0.1 and 0.56 ± 0.2% area in the control and girdled trees, respectively, without a notable treatment effect (Wilcoxon test, *P* = 0.4) and high variability in girdled trees (for individual trees, Table S6 available as Supplementary data at *Tree Physiology* Online). For visualization of histological slices see, Picture S3 available as Supplementary data at *Tree Physiology* Online.

### ^14^C-based estimates of respired CO_2_ age and ^13^C signature of stem-respired CO_2_

**Figure 6:**
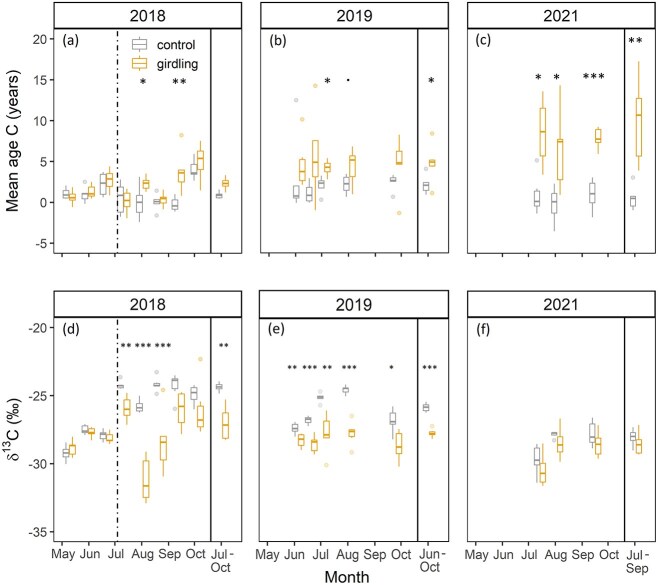
(a–c) Calculated mean age of carbon (^14^C) of the chamber incubation gas samples of control (*n* = 6) and girdled (*n* = 6) poplar trees in (a) 2018, (b) 2019 and (c) 2021. Carbon ages were calculated based on Equ. (2). (d–f) δ^13^C of CO_2_ of the chamber incubation gas samples of control (*n* = 6) and girdled (*n* = 6) poplar trees in (d) 2018, (e) 2019 and (f) 2021. δ^13^C was corrected using Equ. (3). The time of girdling is indicated by the vertical dashed line. Asterisks on top represent the statistical differences between the treatments. Box whisker plots present the median, lower (25th) and upper (75th) percentiles, minimum and maximum values.

Mean age of respired CO_2_ from the pre-girdling sampling was 1.4 years ± 1.1 (control) versus 1.5 years ± 1.3 (girdling) (Figure 6). For the control trees, the C age reached its highest value of 4.0 years ± 1.2 in October 2018, after the leaves had senesced. By contrast, the C age from girdled trees increased up to 15.1 ± 11.8 in 2021. In 2018 and 2019, the C age between the control and girdled trees was significantly different for certain time points with mean differences of all sampling dates in 2019 of 3.5 years and 2021 of 7.5 years (for individual trees, see Tables S4 and S5 available as Supplementary data at *Tree Physiology* Online).

The δ^13^C of CO_2_ (‰) from the pre-girdling sampling was −28.2‰ ± 0.9 (control) versus −28.2‰ ± 0.8 (girdling) (Figure 6). One month after the girdling event, the mean δ^13^C of collected CO_2_ was −25.8‰ ± 0.5 (control) versus −31.2‰ ± 1.6 (girdling). Significant differences between control and girdled trees did occur on specific dates in July and August 2018 and over the whole measurement campaign in 2019. In 2021, the mean δ^13^CO_2_ for the three sampling dates was −28.5‰ ± 1.2 (control) versus −29.2‰ ± 1.4 (girdling) without a notable treatment effect (*P* = 0.15). As a general pattern, the post-girdling δ^13^CO_2_ values of girdled trees were always lower than the δ^13^CO_2_ values of control trees (for individual trees, Table S3 available as Supplementary data at *Tree Physiology* Online) even though, in 2021, the difference was marginal.

The obtained δ^13^C values (±SD) of putative substrates in 2021 were −31.14‰ ± 0.6 (*n* = 12) in neutral lipids and were −27.11‰ ± 0.9 (*n* = 12) in soluble sugars without a treatment effect.

### Potential PEPC activity in woody tissue

At the end of the growing season in 2019, the in vitro PEPC activity (±SD) was 568.2 ± 149.2 and 267.3 ± 94.7 nmol g FW min^−1^ for control and girdled trees, respectively, with a notable treatment effect (*t*-test, *P* < 0.001) (for individual trees, see Table S7 available as Supplementary data at *Tree Physiology* Online).

## Discussion

**Figure 7:**
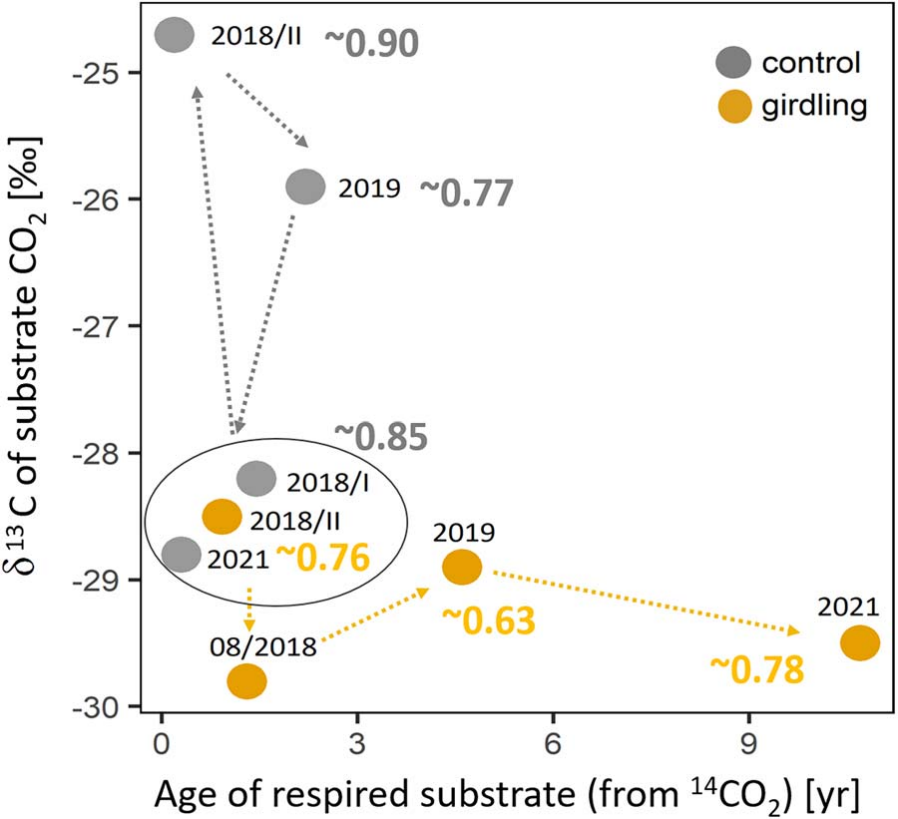
Schematic overview summarizing the results about substrate identity and C mean age. 2018/I refers to pre-girdling and 2018/II refers to post-girdling in 2018. Mean values for the time period June–August (if data available) are shown to avoid seasonal effects. Numbers in bold refer to mean ARQ values. Control trees use fresh photosynthesis products over the study period. After girdling in 2018 and 2019, the δ^13^CO_2_ signal is more enriched, which is possible due to drought effects of those years, while 2021 is comparable with the pre-girdling measurement. ARQ values of ≤0.85 might be explained by a mixture of respiratory substrates, including carbohydrates and lipids. However, ARQ values cannot be seen as a substrate-use indicator alone, as (post-) respiratory processes can affect this ratio (see further explanations in ‘The contribution of lipids during starvation’). In girdled trees, we observed lower δ^13^CO_2_ signal in August 2018 as trees start to use lipids for respiration. After this initial decline, δ^13^CO_2_ signal and ARQ values points toward the use of a mixture of respiratory substrates (COH and lipids). A progressive increase in the mean age of C was observed.

Our 3-year experimental study indicates that the use of a mixture of respiratory substrates with a late contribution of increasingly older reserves provides a mechanism of tree resilience to strong reduction in C supply in poplar trees. Our data suggest that lipid metabolism, indicated by changes in the ^13^C of respired CO_2_ (Figure 7), may allow poplar trees to ride out periods of C starvation; yet, further dedicated studies on lipid metabolism will be helpful. Tree decline may take much longer than the duration of our study, as most of the trees were still alive after the 3-year girdling treatment.

### Significant differences in carbohydrate pools between treatments developed only over time

In our experiment, we combined short- and longer-term responses of poplar trees to a girdling treatment. We could not confirm an initial decrease in NSC concentration (H1), as the concentration in both treatments was very low (starch < 2 mg g^−1^) in 2018 (Figure 5). Girdled trees apparently downregulated their metabolism in concert with sugar supply, as suggested by the strong reduction in respiration rates in 2019 (Figure 3). The downregulation of respiration and growth can be a strategy to maintain certain NSC concentrations in aboveground organs in order to ensure tree survival (Huang et al. 2019). Reduced growth respiration may explain why sugar concentrations initially remained stable. Girdled trees ceased growth after the girdling event (Figure S3, Method S2 available as Supplementary data at *Tree Physiology* Online), which is in accordance with other studies reporting cessation of stem growth below the girdle (Maunoury-Danger et al. 2010, De Schepper and Steppe 2011, Oberhuber et al. 2017) and reduced growth in chilled mature red maple trees below the phloem restriction (Rademacher et al. 2022). Transport of NSC from neighboring trees via root grafts has been shown to be critical for the survival of root suckers in poplar trees (DesRochers and Lieffers 2001, Jelínková et al. 2009); however, net exchange between trees usually is very low (Klein et al. 2016) and may not explain why girdled trees were able to maintain NSC concentrations. In other studies on C limitation, no complete depletion of starch reserves had been observed (e.g., Hoch 2015, Weber et al. 2019) and NSC concentrations of drought-stressed *Picea abies*, also strongly C limited, did not differ to control trees in aboveground organs, whereas only starch reserves in roots strongly declined under drought (Hartmann et al. 2013). The depletion of starch in mature trees under drought stress may take many years (Peltier et al. 2023). However, with regard to stem girdling, various studies showed that NSC concentrations usually decrease below the girdle and/or in the roots, with a concomitant accumulation of NSC above the girdle (Jordan and Habib 1996, Maunoury-Danger et al. 2010, Regier et al. 2010, De Schepper and Steppe 2011, Mei et al. 2015).

In our study, significant differences in carbohydrate pools between treatments developed only over time. In 2021, control trees showed significantly greater carbohydrate concentrations with a 5- to 10-fold increase in starch and soluble sugar, respectively, potentially because climate conditions had normalized after the 2018 and 2019 dry years (see below). Surprisingly, concentrations of soluble sugars in two of the six girdled trees increased in 2021. We hypothesize a remobilization of NSCs from deeper stem layers or from the roots to the section below the girdle in the stem. Overall, the NSC concentrations in girdled trees were more or less stable and remained at a low level over 3 years despite the lack of new photo-assimilate provision. Some of the observed differences between 2018–19 and 2021 may be due to the seasonal variation of NSCs, as sampling dates differed somewhat between years. Concentrations typically decrease after bud break and then increase in the late growing season (Hoch et al. 2003, Richardson et al. 2013, Scartazza et al. 2013, Martínez-Vilalta et al. 2016); however, the seasonal variability of NSCs has been shown to be only 10% in stem sapwood of deciduous trees (Hoch et al. 2003), which was much less than in our study. Also, as our stem cores were not microwaved, this may have resulted in the loss of NSCs to respiration during the initial stage of oven drying (Landhäusser et al. 2018).

### Slow mobilization of older carbohydrate storage pools after girdling

In accordance with our hypothesis (H1), poplar trees accessed older C pools once the supply of fresh assimilates was disrupted. In a girdling study in the Amazon rainforest, trees that were presumably older than 100 years used ~6-year-old C, already 2 months after stem girdling (Muhr et al. 2018), while our ^14^C data indicated a delayed use of older C reserves (Figure 6). Girdled trees respired slightly older C than control trees starting in late summer 2018, except for the date in October, when leaf shedding was almost complete and both treatments used older stored C. In girdled trees, the age of respired CO_2_ increased up to a maximum of 15 years (average 2021: 7.5 years). These values were in accord with previous studies showing maximum a C age of 14 years after girdling of tropical trees (Muhr et al. 2018) and decade-old stored C in temperate trees (Richardson et al. 2013). Control trees relied mostly on recent photo-assimilates throughout the 3-year period (Figure 7). But, mixing of young and old NSCs for stem respiration was also reported in undisturbed mature oak trees (Trumbore et al. 2015).

### The contribution of lipids during starvation

We found evidence supporting our second hypothesis (H2) that poplar trees mobilize and metabolize lipids after starvation via girdling. In August 2018, the δ^13^CO_2_ values close to −30‰ (Figures 6 and 7) indicated that a substantial amount of CO_2_ originated from lipid catabolism (δ^13^C of neutral lipids ~ −32‰, data not shown), but see also ‘Potential effect of the exceptional 2018–19 drought’. We found substantial average differences between treatments in δ^13^CO_2_ (2.5‰), starting already within 1 month after girdling, indicating that control and girdled trees did not use the same respiratory C source mixture. For control trees, we assume that poplar use a mixture of carbohydrates and lipids supporting respiration (Figure 7; mean ARQ: ~0.85, mean δ^13^CO_2_: ~−28.5‰), similar to what have been found in *P. sylvestris* (Fischer et al. 2015). In general, we observed high seasonal variations in control trees (e.g., −29.22‰ (05/2018) to −24.17‰ (08/2018); see Table S3 available as Supplementary data at *Tree Physiology* Online), most likely with increased values during stem growth (Damesin and Lelarge 2003) and seasonal variations due to phenological changes (e.g., leaf growth and senescence) (Seibt et al. 2008).

The observed decline in ARQ values after the girdling treatment in autumn 2018 was followed by a more drastic decline in the ARQ values during the summer months in 2019 (~0.63, Figures 3 and 7), indicating the contribution of lipids to maintain metabolism during starvation. During shading-induced starvation, Fischer et al. (2015) found evidence for lipid metabolism, with ARQ ratios of ~0.7 in *P. sylvestris* trees. In May/June (2018 and 2019), we observed the highest ARQ, which could indicate the use of carbohydrates caused by mobilization of reserves. The treatment effect in ARQ and δ^13^CO_2_ decreased then toward the end of the seasons, likely because the remobilization of storage decreased at the onset of dormancy. Despite the high soluble sugar and starch concentrations observed in control trees in 2021, we found no clear difference in δ^13^CO_2_ or ARQ in that year. By contrast, we observed with the histological staining method (Table S6 available as Supplementary data at *Tree Physiology* Online) the depletion of lipids in one of the girdled trees in 2021, but dedicated studies to address the lipid distribution in stem wood would be necessary to fully understand lipid metabolism in stems under C limitation.

The ARQ is a useful indicator for respiratory substrate use, but a variety of other processes, like stem photosynthesis, CO_2_ transport in the sap or CO_2_ refixation, can lead to <1 ARQ value (see Trumbore et al. 2013 for a summary). In our study, we can neglect stem photosynthesis because we used opaque chambers. Stem photosynthesis around the chamber could generate a gradient of CO_2_ and cause axial diffusion of CO_2_ from the chamber to the illuminated parts, affecting ARQ (De Roo et al. 2019). Also, high solubility of CO_2_ in the xylem sap may facilitate import to or export from the site of measurement (Teskey and McGuire 2007, Aubrey and Teskey 2009, Bloemen et al. 2013). Considering that sap flow was reduced in girdled trees in 2018 (Figure S1 available as Supplementary data at *Tree Physiology* Online), indicating a limited amount of upward transport, we would expect a correlation between the sap flow and absolute deviation of ARQ from unity. Refixation of CO_2_ via the enzyme PEPC might remove CO_2_ locally, but we found ~50% reduced PEPC activity in girdled trees the year after girdling (Table S7 available as Supplementary data at *Tree Physiology* Online), which suggests a limited role of PEPC-mediated C fixation in lowering the ARQ values.

### Potential effect of the exceptional 2018–19 drought

Our study coincided with the exceptionally dry and hot summers in 2018 and 2019 in Central Europe during which C supply may have been impaired also in control trees from reduced C assimilation. A tree ring analysis showed ~50% reduced tree ring width in 2019, but also in 2020, which can be seen as a legacy effect of drought (e.g., Miller et al. 2023) (Figure S3 available as Supplementary data at *Tree Physiology* Online). Meanwhile in 2018, trees either were still able to cope with the dry and hot conditions, or were simply not yet severely affected by the 2018 summer drought at the north slope where our study was located. The *E*_CO2_ and *I*_O2_ were reduced by ~40% in 2019 compared with 2018 in control trees, which could potentially be explained by the drought effect in 2019. Similar results (50% decline in *E*_CO2_) were observed in *Quercus ilex* when the soil predawn water potential decreased (Rodríguez-Calcerrada et al. 2014). Drought-induced decline in stomatal conductance would also lead to increased δ^13^C values of CO_2_ (due to changes in photosynthetic discrimination; Farquhar et al. 1989, Högberg et al. 1995), as most likely observed in 2018 and 2019. Compared with wetter years, an increase in the δ^13^CO_2_ of control trees might also be due to starch hydrolysis as an alternative and more enriched C source that causes a shift toward a more enriched CO_2_ pool (Maunoury-Danger et al. 2010). In 2021, control trees showed no longer an enriched δ^13^C, possibly because fresh C no longer had a drought signal in δ^13^C. Overall, we critically note that we did not assess tree water relations and that we lack evidence of how severe the drought stress was.

### Conclusion

Storage use provides a buffer and enables long-term survival under periods when C supply from the canopy is not given. Insights about how long trees can store and access reserve compounds in response to changes in source-sink relationships are highly needed to improve our understanding of tree resilience under ongoing climate change. Several studies have highlighted the shortcomings in the representation of tree stress responses and resilience, mediated by C storage, in vegetation models (Ogle and Pacala 2009, DeSoto et al. 2020, Peltier and Ogle 2020, Hartmann et al. 2022). The common assumption that assimilated C via photosynthesis is directly respired to the atmosphere needs to be updated for model improvement (Sierra et al. 2022) as it is contradictory to our study results and previous empirical evidence (Vargas et al. 2009, Carbone et al. 2013, Muhr et al. 2013, 2018). Combining empirical studies on the remobilization and metabolization of C with C dynamic models may help improve predictions and constrain model parameters (Sierra et al. 2022).

## Supplementary Material

Supplementary_Data_tpad135

## Data Availability

The data that support the findings of this study are available from the corresponding author upon request.
