# Supplementary material for: Carbon dynamics in long-term starving poplar trees—the importance of older carbohydrates and a shift to lipids during survival
Source: Tree Physiol. 2023 Nov 6;44(13):173–85. doi: 10.1093/treephys/tpad135 (PMC11898624; doi:10.1093/treephys/tpad135)
Supplement: Supplementary_Data_tpad135 [file supplementary_data_tpad135.docx]

# **Supplementary Data**

Carbon dynamics during long-term survival of starving poplar trees - an initial shift to lipids followed by the use of older carbohydrates

Authors: Juliane Helm, Jan Muhr, Boaz Hilman, Ansgar Kahmen, Ernst-Detlef Schulze, Susan Trumbore, David Herrera-Ramírez, Henrik Hartmann

The following Supporting Information is available for this article:

**Table S1** Diameter at breast height (cm) of the 12 poplar trees (6 control, 6 girdled) in July 2018. Volume of the stem chamber was determined once after installation in each year.

**Table S2** Overview of measurements taken in all three campaigns 2018, 2019 and 2021 or in individual years, only.

**Table S3** Mean δ^13^C of CO_2_ (±SD) of the chamber incubation gas samples for all trees individually.

**Table S4** Mean Δ^14^C (±SD) of the chamber incubation gas samples for all trees individually.

**Table S5** Mean Age of C (±SD) of the chamber incubation gas samples for all trees individually.

**Table S6** Neutral lipid quantification for control and girdled trees in 2021 (sampling, DOY 147). First 3 cm has been analyzed.

**Table S7** In vitro PEPC activity for control and girdled trees in 2019 (from wood material of the first 2 cm, sampling: DOY 236).

**Picture S1** a) Two girdled and one control poplar tree at the study site in September 2018, b) girdling band in 2018, c) control tree in 2021, d) girdling band in 2021 and e) girdled tree in 2021.

**Picture S2** Experimental set-up with the respiration chamber below the girdling, the glass flasks for gas sample analysis and Sap Flow Meter SFM1 in 2018.

**Picture S3** Wood histological slices stained with Oil Red O (ORO) (C_26_H_24_N_4_O). This is a lysochrome (fat-soluble dye) used for staining neutral lipids (i.e. triglycerides, diacylglycerols and cholesterol esters). We visualized neutral lipids in a) a control, b) a girdled tree and c) red lipid droplets in ray parenchyma cells of a control tree.

**Figure S1** Sap flow rate (l h^-1^) in 2018 (n = 6 for control (grey) and girdled (yellow) trees, respectively). Girdling event took place 4^th^ of July 2018.

**Figure S2** Soluble sugar (glucose, fructose, sucrose) concentration (mg g^-1^) and starch concentration (mg g^-1^) before girdling (DOY 172) and three time points after girdling, extracted from stem cores from a depth of ring 6 to 14. Colors denote treatment (n = 6 for control (grey) and girdled (yellow) trees, respectively). Box whisker plots present the median, lower (25^th^) and upper (75^th^) percentiles, minimum and maximum values.

**Figure S3** Deviation of tree ring width (TRW) from the 20 yr average (TRW_2002-2022_), analyzed in control trees (grey; n = 3) and girdled trees, respectively (yellow; n = 3).

**Method S1** Estimation of the δ^13^C signatures of sugars and neutral lipids.

**Method S2** Tree ring width analysis.

**Table S1** Diameter at breast height (cm) of the 12 poplar trees (6 control, 6 girdled) in July 2018. Volume of the stem chamber was determined once after installation in each year.

| **Tree Number** | **DBH (cm)** | **Treatment** | **Volume determination (cm^3^)** | | |
| --- | --- | --- | --- | --- | --- |
|  |  |  | 2018 | 2019 | 2021 |
| 1 | 36.0 | Girdling | 92 |  |  |
| 2 | 39.3 | Control | 101 |  |  |
| 3 | 34.6 | Control | 105 | 84 | 95 |
| 4 | 32.8 | Control | 96 |  |  |
| 5 | 30.4 | Control | 98 | 84 | 98 |
| 6 | 31.1 | Girdling | 96 |  | 100 |
| 7 | 29.1 | Girdling | 83 |  | 93 |
| 8 | 33.4 | Girdling | 99 | 88 | 90 |
| 9 | 41.9 | Control | 101 | 97 | 87 |
| 10 | 37.1 | Girdling | 104 | 85 | 97 |
| 11 | 34.9 | Girdling | 99 | 97 |  |
| 12 | 36.3 | Control | 76 |  | 92 |

**Table S2** Overview of measurements taken in all three campaigns 2018, 2019 and 2021 or in individual years, only.

| **Measurement** | **Time period** | **Number of trees (n)** |
| --- | --- | --- |
| CO_2_ efflux, O_2_ influx | 2018, 2019, 2021 | 12, 6, 8 |
| NSC | 2018 (DOY 172, 262), 2019 (DOY 147), 2021 (DOY 195) | 10-12 (when analysis failed) |
| δ^13^CO_2_,^14^C | 16 measurement points, for details see Tables S4, S6 | 10-12 (when analysis failed) |
| Sap flow | 2018 | 12 |
| PEPC activity | 2019 (DOY 236) | 11 |
| Neutral lipids (staining method) | 2021 (DOY 147) | 6 |
| δ^13^C of substrates | 2021 (DOY 236) | 12 |

**Table S3** Mean δ^13^C of CO_2_ (±SD) of the chamber incubation gas samples for all trees individually.

| **Year** | **Date** | **DOY** | **Treatment** | | **Sample Number (n)** | |
| --- | --- | --- | --- | --- | --- | --- |
|  |  |  | **Control ± SD** | **Girdled ± SD** | **Control** | **Girdled** |
| 2018 | 09.05.2018 | 129 | -29.22 ± 0.6 | -28.84 ± 0.6 | 6 | 6 |
|  | 31.05.2018 | 151 | -27.50 ± 0.3 | -27.70 ± 0.4 | 6 | 6 |
|  | 21.06.2018 | 172 | -27.90 ± 0.4 | -28.11 ± 0.4 | 6 | 6 |
|  | 11.07.2018 | 192 | -24.23 ± 0.3 | -25.93 ± 0.9 | 6 | 6 |
|  | 02.08.2018 | 214 | -25.78 ± 0.5 | -31.20 ± 1.6 | 6 | 6 |
|  | 22.08.2018 | 234 | -24.17 ± 0.5 | -28.40 ± 2.2 | 6 | 6 |
|  | 12.09.2018 | 255 | -24.27 ± 0.9 | -26.04 ± 1.5 | 6 | 4 |
|  | 04.10.2018 | 277 | -24.92 ± 0.7 | -26.07 ± 2.0 | 6 | 6 |
| 2019 | 05.06.2019 | 156 | -27.37 ± 0.4 | -28.26 ± 0.5 | 6 | 6 |
|  | 20.06.2019 | 171 | -26.76 ± 0.3 | -28.51 ± 0.6 | 6 | 6 |
|  | 04.07.2019 | 185 | -25.09 ± 0.3 | -30.92 ± 7.8 | 6 | 5 |
|  | 02.08.2019 | 214 | -24.51 ± 0.3 | -27.71 ± 0.9 | 6 | 6 |
|  | 25.09.2019 | 268 | -26.80 ± 0.9 | -28.65 ± 1.0 | 5 | 6 |
| 2021 | 14.07.2021 | 195 | -29.70 ± 1.1 | -30.47 ± 1.2 | 6 | 6 |
|  | 03.08.2021 | 215 | -27.85 ± 0.2 | -28.48 ± 1.1 | 6 | 6 |
|  | 16.09.2021 | 259 | -27.81 ± 0.9 | -28.57 ± 0.9 | 6 | 6 |

**Table S4** Mean Δ^14^C (±SD) of the chamber incubation gas samples for all trees individually.

| **Year** | **Date** | **DOY** | **Treatment** | | **Sample Number (n)** | |
| --- | --- | --- | --- | --- | --- | --- |
|  |  |  | **Control ± SD** | **Girdled ± SD** | **Control** | **Girdled** |
| 2018 | 09.05.2018 | 129 | 7.28 ± 1.2 | 5.25 ± 1.6 | 6 | 6 |
|  | 31.05.2018 | 151 | 6.87 ± 1.8 | 8.48 ± 1.5 | 6 | 6 |
|  | 21.06.2018 | 172 | 12.77 ± 2.7 | 15.15 ± 2.5 | 6 | 6 |
|  | 11.07.2018 | 192 | 4.70 ± 3.7 | 2.98 ± 2.6 | 6 | 6 |
|  | 02.08.2018 | 214 | 2.53 ± 3.8 | 13.17 ± 1.6 | 6 | 6 |
|  | 22.08.2018 | 234 | 2.47 ± 1.9 | 3.93 ± 1.6 | 6 | 6 |
|  | 12.09.2018 | 255 | 1.20 ± 1.6 | 19.90 ± 4.8 | 6 | 6 |
|  | 04.10.2018 | 277 | 21.22 ± 2.3 | 25.73 ± 4.1 | 6 | 6 |
| 2019 | 05.06.2019 | 156 | 12.44 ± 11.1 | 19.42 ± 5.7 | 5 | 6 |
|  | 20.06.2019 | 171 | 3.05 ± 3.1 | 24.80 ± 14.8 | 4 | 4 |
|  | 04.07.2019 | 185 | 7.33 ± 2.9 | 17.40 ± 2.5 | 4 | 4 |
|  | 02.08.2019 | 214 | 7.98 ± 2.1 | 18.38 ± 4.2 | 6 | 6 |
|  | 25.09.2019 | 268 | 9.37 ± 1.8 | 19.55 ± 6.2 | 6 | 6 |
| 2021 | 14.07.2021 | 195 | -1.33 ± 4.5 | 66.98 ± 23.1 | 6 | 6 |
|  | 03.08.2021 | 215 | -6.55 ± 4.1 | 25.23 ± 9.5 | 6 | 6 |
|  | 16.09.2021 | 259 | -0.92 ± 3.5 | 31.42 ± 2.8 | 6 | 5 |

**Table S5** Mean Age of C (±SD) of the chamber incubation gas samples for all trees individually.

| **Year** | **Date** | **DOY** | **Treatment** | | **Sample Number (n)** | |
| --- | --- | --- | --- | --- | --- | --- |
|  |  |  | **Control ± SD** | **Girdled ± SD** | **Control** | **Girdled** |
| 2018 | 09.05.2018 | 129 | 1.06 ± 0.6 | 0.63 ± 0.8 | 6 | 6 |
|  | 31.05.2018 | 151 | 0.97 ± 0.9 | 1.32 ± 0.8 | 6 | 6 |
|  | 21.06.2018 | 172 | 2.23 ± 1.4 | 2.73 ± 1.3 | 6 | 6 |
|  | 11.07.2018 | 192 | 0.51 ± 1.9 | 0.15 ± 1.3 | 6 | 6 |
|  | 02.08.2018 | 214 | 0.05 ± 1.9 | 2.31 ± 0.8 | 6 | 6 |
|  | 22.08.2018 | 234 | 0.04± 1.0 | 0.35 ± 0.8 | 6 | 6 |
|  | 12.09.2018 | 255 | -0.23 ± 0.8 | 3.74 ± 2.5 | 6 | 6 |
|  | 04.10.2018 | 277 | 4.02 ± 1.2 | 4.99 ± 2.2 | 6 | 6 |
| 2019 | 05.06.2019 | 156 | 3.16± 5.3 | 4.64 ± 3.0 | 5 | 6 |
|  | 20.06.2019 | 171 | 1.16 ± 1.3 | 5.79 ± 6.3 | 4 | 4 |
|  | 04.07.2019 | 185 | 2.07 ± 1.2 | 4.21 ± 1.1 | 4 | 4 |
|  | 02.08.2019 | 214 | 2.21 ± 1.1 | 4.42 ± 2.2 | 6 | 6 |
|  | 25.09.2019 | 268 | 2.50 ± 0.9 | 4.67 ± 3.2 | 6 | 6 |
| 2021 | 14.07.2021 | 195 | 0.87 ± 2.3 | 15.4 ± 12.0 | 6 | 6 |
|  | 03.08.2021 | 215 | -0.24 ± 2.1 | 6.52 ± 5.0 | 6 | 6 |
|  | 16.09.2021 | 259 | 0.95 ± 1.8 | 7.83 ± 1.3 | 6 | 5 |

**Table S6** Neutral lipid quantification for control and girdled trees in 2021 (sampling, DOY 147). First 3 cm has been analyzed.

| Treatment | Tree number | Lipid quantification (% area)  (first 3 cm) |
| --- | --- | --- |
| Control | 3 | 0.72 |
|  | 9 | 0.89 |
|  | 12 | 0.67 |
| Girdling | 7 | 0.61 |
|  | 8 | 0.75 |
|  | 10 | 0.33 |

**Table S7** In vitro PEPC activity for control and girdled trees in 2019 (from wood material of the first 2 cm, sampling: DOY 236).

| Treatment | Tree number | PEPC activity (nmol/gFW/min^-1^) |
| --- | --- | --- |
| Control | 2 | 764.8.0 |
|  | 3 | 529.1 |
|  | 4 | 417.7 |
|  | 5 | 398.0 |
|  | 9 | 594.7 |
|  | 12 | 705.2 |
| Girdling | 1 | NA |
|  | 6 | 129.1 |
|  | 7 | 257.2 |
|  | 8 | 241.1 |
|  | 10 | 334.4 |
|  | 11 | 374.4 |

***
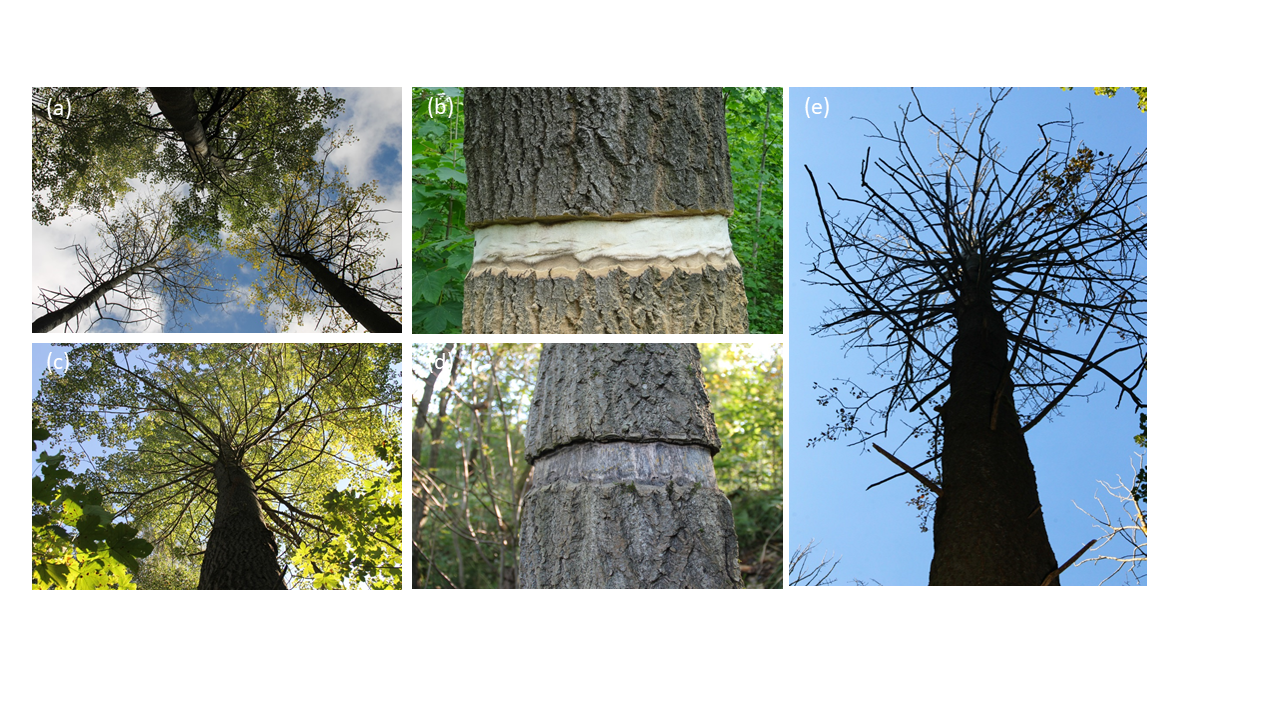
***

**Picture S1** a) Two girdled and one control poplar tree at the study site in September 2018, b) girdling band in 2018, c) control tree in 2021, d) girdling band in 2021 and e) girdled tree in 2021.


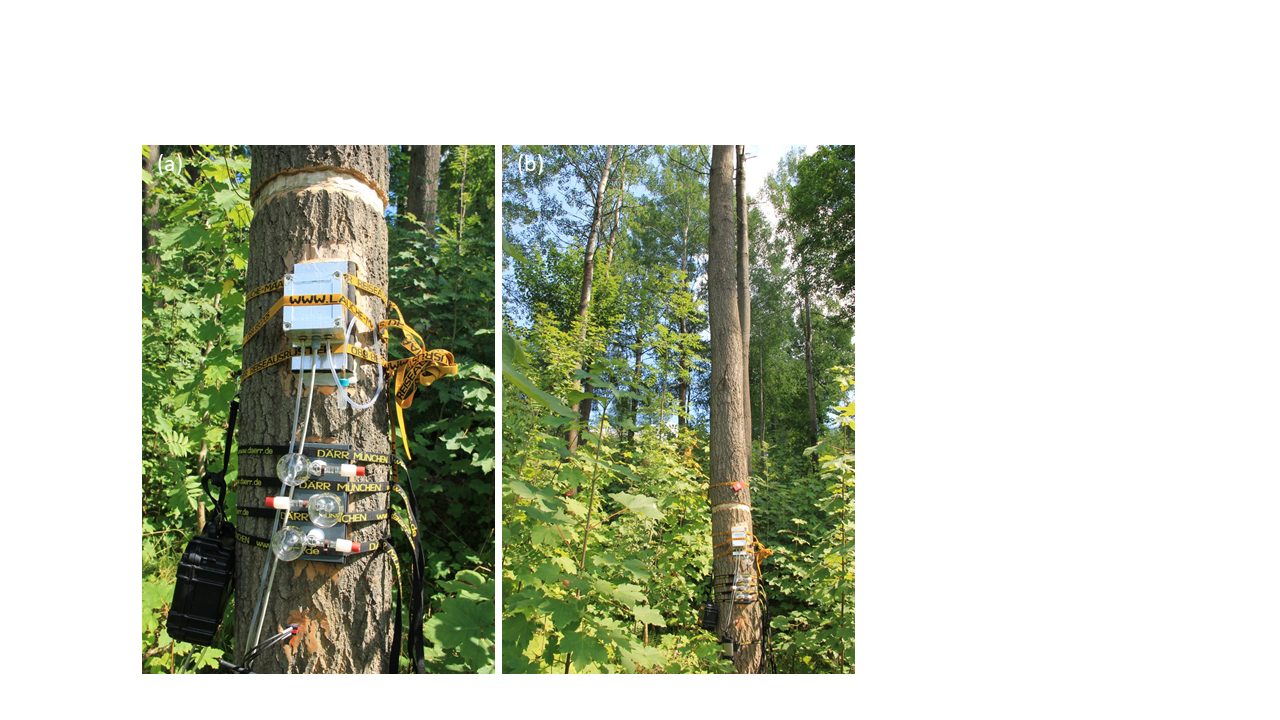


**Picture S2** Experimental set-up with the respiration chamber below the girdling, the glass flasks for gas sample analysis and Sap Flow Meter SFM1 in 2018.

| 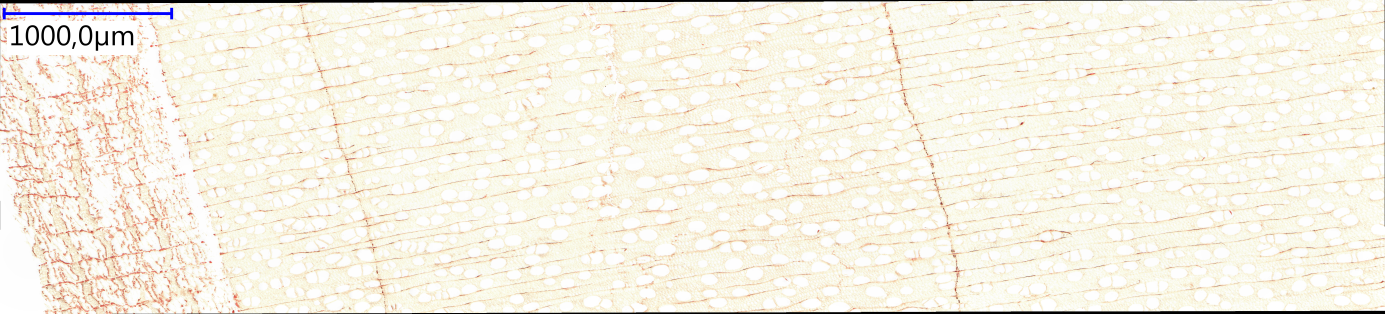  (a)  (b)  (c) |
| --- |
| 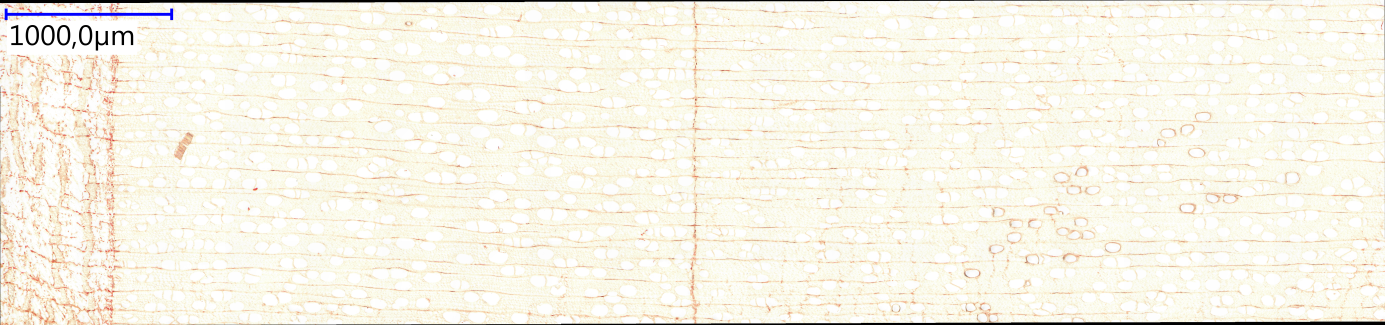 |
| 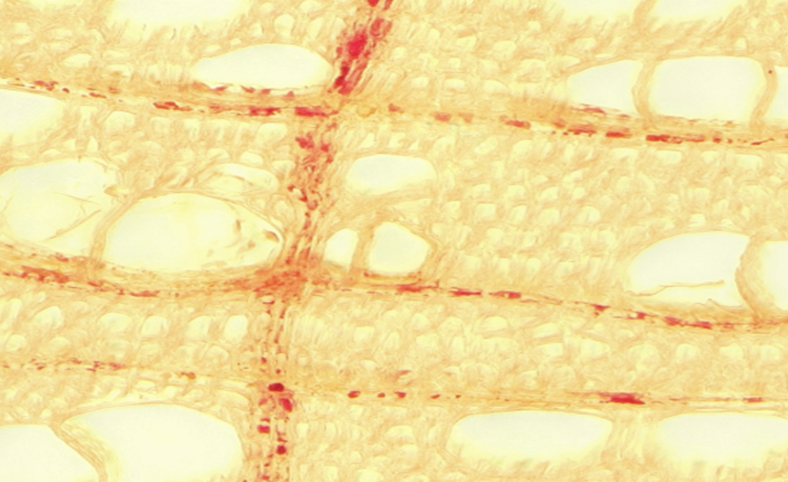 |

**Picture S3** Wood histological slices stained with Oil Red O (ORO) (C_26_H_24_N_4_O). This is a lysochrome (fat-soluble dye) used for staining neutral lipids (i.e. triglycerides, diacylglycerols and cholesterol esters). We visualized neutral lipids in a) a control, b) a girdled tree and c) red lipid droplets in ray parenchyma cells of a control tree.


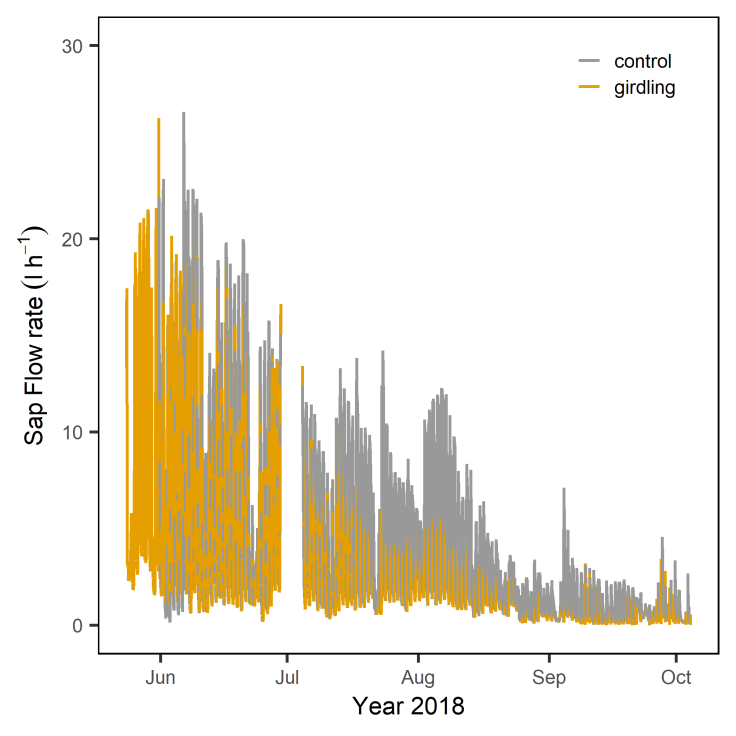


**Figure S1** Sap flow rate (l h^-1^) in 2018 (n = 6 for control (grey) and girdled (yellow) trees, respectively). Girdling event took place 4^th^ of July 2018.


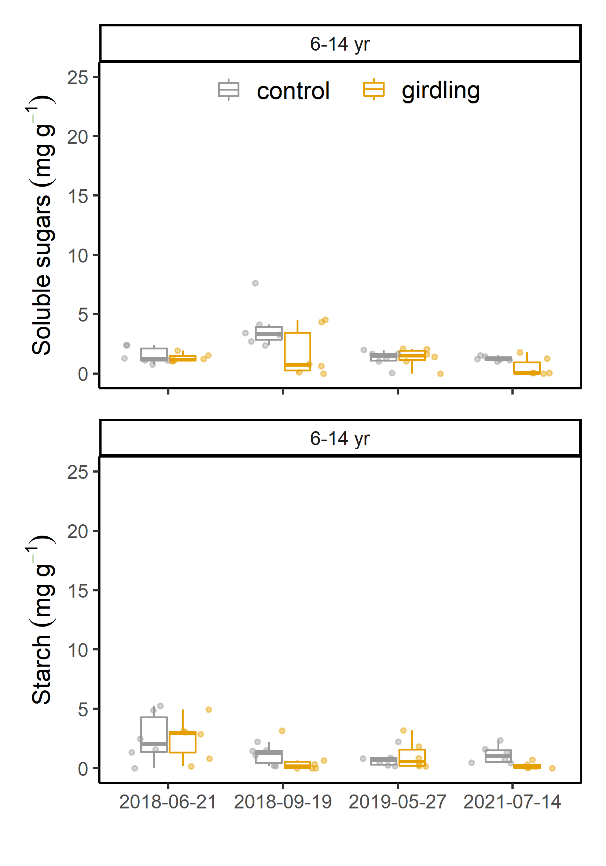


**Figure S2** Soluble sugar (glucose, fructose, sucrose) concentration (mg g^-1^) and starch concentration (mg g^-1^) before girdling (DOY 172) and three time points after girdling, extracted from stem cores from a depth of ring 6 to 14. Colors denote treatment (n = 6 for control (grey) and girdled (yellow) trees, respectively). Box whisker plots present the median, lower (25^th^) and upper (75^th^) percentiles, minimum and maximum values.

**
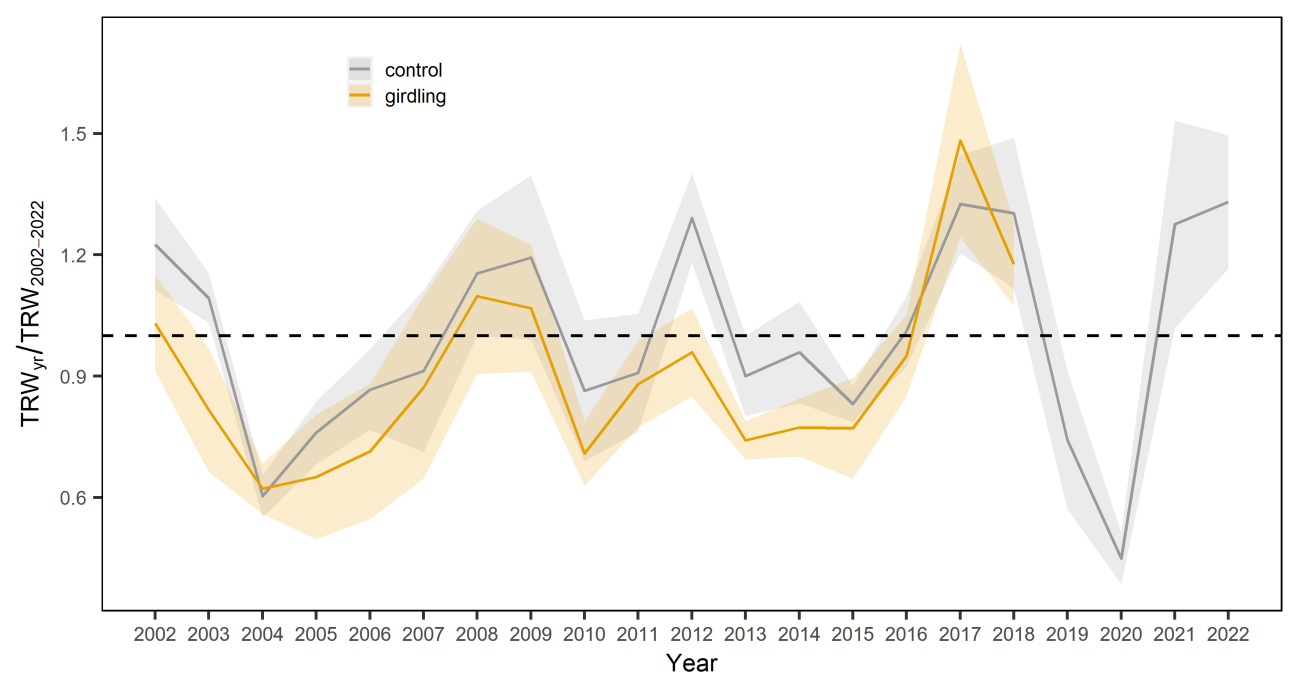
**

**Figure S3** Deviation of tree ring width (TRW) from the 20 yr average (TRW_2002-2022_), analyzed in control trees (grey; n = 3) and girdled trees (yellow; n = 3).

**Method S1** Estimation of the δ^13^C signatures of sugars and neutral lipids.

To estimate the δ^13^C signatures of sugars and neutral lipids we followed a modified protocol (Bligh and Dyer 1959; White et al. 1979) and liquid chromatography (Schwab et al. 2019). We separated stem cores into the two treatments (n = 6, each). We then milled the wood and prepared 100 mg aliquots, sonicated in a mixture of chloroform, methanol and water, and centrifuged at 2000 rpm for 10 min. The upper phase in the solution contains the water and its solutes, e.g. sugars. To exhaust all sugars we re-added water, centrifuged, and collected the water phase two more times. The lower phase that assumed to contain all lipid fractions was dried over sodium sulfate. Then we extracted the neutral-lipids fraction with column chromatography (SPE 6 ml column) of silica gel (Merck silica mesh 230–400, 3 g activated 1 hr at 120 °C) eluted with 18 ml chloroform. Aliquotes from the sugars and neutral lipids solutions were pipetted into tin capsules and analyzed for δ13C in Delta+XL IRMS (Thermo Finnigan, Bremen, Germany).

Bligh, E.G. and W.J. Dyer. 1959. A rapid method of total lipid extraction and purification. Canadian journal of biochemistry and physiology. 37:911-917.

Schwab, V.F., M.E. Nowak, S.E. Trumbore, X. Xu, G. Gleixner, J. Muhr, K. Küsel and K.U. Totsche. 2019. Isolation of individual saturated fatty acid methyl esters derived from groundwater phospholipids by preparative high‐pressure liquid chromatography for compound‐specific radiocarbon analyses. Water Resources Research. 55:2521-2531.

White, D., W. Davis, J. Nickels, J. King and R. Bobbie. 1979. Determination of the sedimentary microbial biomass by extractible lipid phosphate. Oecologia. 40:51-62.

**Method S2** Tree ring width analysis.

In order to quantify radial growth and to better evaluate whether the drought events in 2018 and 2019 impacted our measurements in the control trees, we analyzed variations in tree ring width as an indicator of stress. In 2022, we took increment cores from 3 control trees and 3 girdled trees (west side of the trees). After gluing the cores on wood strips, we cut them with a core-microtome using a cutter blade (Gärtner and Nievergelt 2010). To enhance contrast for analysis of ring boundaries, the cut surface was stained with ink, and covered with white chalk powder to fill vessels of earlywood (González-González et al. 2014). Stem cores were scanned at 2400 dpi. Radial growth measurements and cross-dating were performed using the program CDendro suite software (Cybis Elektronik & Data, Saltsjöbaden, Sweden). Cross-dating was further checked by comparing the tree-ring series to that of non-girdled spruces trees growing near to the site. We calculated the 20-yr average ring width (2002-2022) and compared this calculated average with each individual year for control and girdled trees, respectively.

Gärtner, H. and D. Nievergelt. 2010. The core-microtome: a new tool for surface preparation on cores and time series analysis of varying cell parameters. Dendrochronologia. 28:85-92.

González-González, B.D., V. Rozas and I. García-González. 2014. Earlywood vessels of the sub-Mediterranean oak Quercus pyrenaica have greater plasticity and sensitivity than those of the temperate Q. petraea at the Atlantic–Mediterranean boundary. Trees. 28:237-252.
